# Post-exertional malaise in Long COVID: subjective reporting versus objective assessment

**DOI:** 10.3389/fneur.2025.1534352

**Published:** 2025-04-23

**Authors:** Barbara Stussman, Nathan Camarillo, Gayle McCrossin, Marybeth Stockman, Gina Norato, C. Stephenie Vetter, Alenka Ferrufino, Ashade Adedamola, Nicholas Grayson, Avindra Nath, Leighton Chan, Brian Walitt, Lisa M. K. Chin

**Affiliations:** ^1^National Institute of Neurological Disorders and Stroke, National Institutes of Health, Bethesda, MD, United States; ^2^Rehabilitation Medicine Department, National Institutes of Health Clinical Center, Bethesda, MD, United States

**Keywords:** post-COVID condition, post-exertional symptom exacerbation, post acute sequelae of SARS-CoV-2, cardiopulmonary exercise test (CPET), myalgic encephalomyelitis/chronic fatigue syndrome (ME/CFS)

## Abstract

**Background:**

Post-exertional malaise (PEM) is a central feature of myalgic encephalomyelitis/chronic fatigue syndrome (ME/CFS) and has emerged as a prominent feature of Long COVID. The optimal clinical approach to PEM is inconclusive, and studies of the impact of exercise have yielded contradictory results.

**Objective:**

The objective of this study was to examine PEM in Long COVID by assessing the prevalence of self-reported PEM across study cohorts and symptom responses of Long COVID patients to a standardized exercise stressor. Secondarily, Long COVID symptom responses to exercise were compared to those of ME/CFS and healthy volunteers.

**Methods:**

Data from three registered clinical trials comprised four cohorts in this study: Long COVID Questionnaire Cohort (QC; *n* = 244), Long COVID Exercise Cohort (EC; *n* = 34), ME/CFS cohort (*n* = 9), and healthy volunteers (HV; *n* = 9). All cohorts completed questionnaires related to physical function, fatigue, and/or PEM symptoms. EC also performed a standardized exercise test (cardiopulmonary exercise test, CPET), and the PEM response to CPET was assessed using visual analog scales and qualitative interviews (QIs) administered serially over 72 h. EC PEM measures were compared to ME/CFS and HV cohorts. A secondary analysis of QI explored positive responses to CPET among EC, ME/CFS and HV.

**Results:**

Self-reported PEM was 67% in QC and estimated at 27% in EC. Only 2 of 34 EC patients (5.9%) were observed to develop PEM after a CPET. In addition, PEM responses after CPET in Long COVID were not as severe and prolonged as those assessed in ME/CFS. Twenty-two of 34 EC patients (64.7%) expressed at least one of 7 positive themes after the CPET.

**Conclusion:**

Self-report of PEM is common in Long COVID. However, observable PEM following an exercise stressor was not frequent in this small cohort. When present, PEM descriptions during QI were less severe in Long COVID than in ME/CFS. Positive responses after an exercise stressor were common in Long COVID. Exercise testing to determine the presence of PEM may have utility for guiding clinical management of Long COVID.

## 1 Introduction

Long COVID is described as persistent disabling symptoms following infection with SARS-CoV-2 and more recently has been defined as a chronic condition presenting 3 months or longer after COVID infection, with symptoms manifesting in one or multi-organ systems (1). A wide variety of symptoms and conditions have been associated with Long COVID (such as severe and persistent fatigue that may relate to poor sleep quality and cognitive function, and postural orthostatic tachycardia syndrome (POTS) reflecting autonomic dysfunction), which further impacts physical functioning and reduces quality of life (2). Post-exertional malaise (PEM) is described as acute worsening of disabling symptoms such as severe fatigue, low exercise tolerance, and cognitive issues, following minimal physical or mental exertion (3). While PEM is a central feature of myalgic encephalomyelitis/chronic fatigue syndrome (ME/CFS) (4), it has also more recently emerged as a feature of Long COVID (1, 5–7). Often described as all-encompassing and necessitating complete bedrest for recovery, the PEM experience can be hard to manage and predict. Common symptoms of PEM in ME/CFS include physical fatigue, cognitive difficulties, neuromuscular complaints, and sleep disturbances (8, 9). The features of PEM are often not immediate and can extend days beyond the exertion that triggered the responses, with peaks observed within hours to days (8–10). Under research conditions, cardiopulmonary exercise testing (CPET) is an important assessment for inducing and evaluating the presence of PEM (11, 12).

Two nationally representative surveys put the estimate of U.S. adults with Long COVID at 6.9 and 6.4%, respectively (13, 14), but robust national data on the prevalence of PEM in Long COVID are lacking. The DePaul Symptom Questionnaire (DSQ), originally developed as a 99-item questionnaire to assess various aspects of ME/CFS (15), has demonstrated strong reliability and validity (16, 17). Since its initial development, shorter 14-item and 5-item brief questionnaires have been developed and tested (18, 19). An evaluation for sensitivity and specificity of the 5-item DSQ demonstrated its ability to differentiate PEM in ME/CFS from other fatiguing conditions (19). The items correctly categorized ME/CFS patients 81.7% of the time while incorrectly categorizing patients with other fatiguing conditions as having ME/CFS only 16.6% of the time. More recently, the DSQ has been used to evaluate PEM in Long COVID. Two studies (20, 21) reported 59% of adults post-SARS-CoV−2 infection met the threshold for PEM by DSQ, while another study of outpatients testing positive for SARS-CoV-2 determined 8.2% had PEM at a 6-month follow-up (22). Another study found that nearly 30% of Long COVID patients reported fatigue after exertion lasting more than 24 h (23). Clearly, more information about the prevalence and severity of PEM and its impact on physical function in Long COVID is needed.

The role of physical activity in Long COVID has similarly yielded conflicting results. A notable study sought to induce PEM in 25 Long COVID patients using a maximal exercise test and found a tissue damage response compared to healthy controls (6), while worsening of symptoms after engaging in physical activity was reported among those surveyed with Long COVID (24). Yet despite these negative observations, recent literature has highlighted the benefits of exercise in Long COVID and the risks of guidelines urging caution (25–27). A recent study found that Long COVID patients who took part in Nordic Walking sessions over the course of 3 months experienced a decrease in fatigue and improved quality of life (28). Other studies have similarly reported improved PEM in Long COVID patients following progressive or tailored exercise-programming (29, 30). Several other studies have also found no worsening fatigue (31–33) among Long COVID patients when compared to healthy controls or a non-exercising group following exercise programs. These conflicting data suggest that a better understanding of the relationship between exercise, PEM, and outcomes could have a clinical impact on the approach to Long COVID treatment.

It is also not clear if self-reported PEM in Long COVID is the same as the PEM reported in ME/CFS. The limited research comparing PEM in Long COVID versus ME/CFS has found both groups experience similar PEM symptoms except ME/CFS patients report unrefreshed sleep and flu-like symptoms more frequently and Long COVID patients tend to report more respiratory symptoms (7, 34). These studies found no differences in symptom severity, onset, and duration of PEM between the groups. Another recent study also found no differences in PEM severity between Long COVID and ME/CFS (35). More recently, Unger et al. (36) found that in a large cohort study, 3.4% of persons who had SARS-CoV-2 met the Institute of Medicine (IOM) criteria for ME/CFS using the CDC ME/CFS Symptom Screener-Short Form (4).

The current study sought to add to existing literature by (1) examining the prevalence of self-reported PEM among individuals with Long COVID, (2) examining PEM responses to a standardized exercise stressor among individuals with Long COVID, and (3) comparing PEM among individuals with Long COVID versus ME/CFS.

## 2 Methods

### 2.1 Study design and cohorts

**Figure 1:**
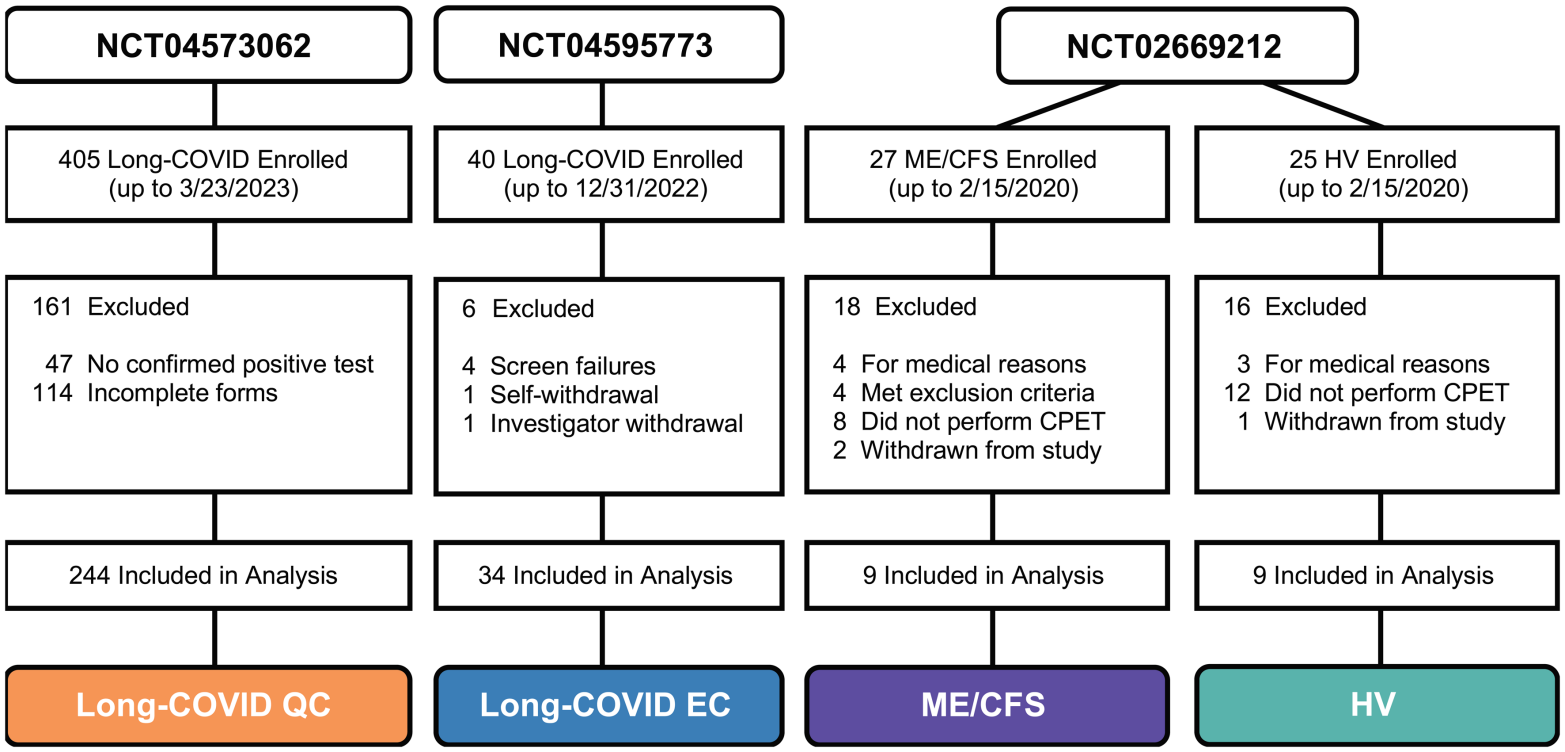
Patient flow and cohort by clinical trial study.

The current study employed a two-part study design utilizing data across three registered clinical trials. Data were compiled on Long COVID patients’ assessments of PEM using self-reported questionnaire data (NCT04573062), while measures of PEM after undergoing CPET were analyzed in Long COVID (NCT04595773), ME/CFS, and HV patients (NCT02669212). Therefore, the study population of the current study included four cohorts: Long COVID Questionnaire Cohort (QC; *n* = 244), Long COVID Exercise Cohort (EC; *n* = 34), ME/CFS cohort (*n* = 9), and healthy volunteers (HV; *n* = 9). All cohorts were mutually exclusive except for six patients who were co-enrolled in QC and EC. We used our recently described mixed method system for effective objective assessment of PEM among ME/CFS (37) by performing qualitative interviews (QIs) at multiple timepoints before and after a CPET in conjunction with questionnaires and physical measurements. QC patients completed a battery of online questionnaires describing a wide range of Long COVID-related symptoms. In addition to filling in questionnaires, the EC, ME/CFS, and HV cohorts underwent a cardiopulmonary exercise test (CPET) and were evaluated for PEM through serial qualitative interviews (QIs) and visual analog scale (VAS). Self-assessments were captured by questionnaires, while performance of the CPET in combination with QIs allowed objective assessment of PEM by the research team. The six Long COVID patients who took part in both the QC and EC studies allowed for individual tracking across study participation. All studies were approved by the Institutional Review Board at the National Institutes of Health (NIH) and registered on clinicaltrials.gov. Written consent was obtained from all patients prior to formal enrollment and study participation. The flow of patients from all studies is presented in Figure 1.

### 2.2 Recruitment

Recruitment for the QC began in July 2020 and was conducted primarily via self-referral through the NIH patient recruitment office, the NIH Clinical Trial web page, the US clinical trials registry,^1^ and clinician referrals for patients with concern for Long COVID. Completed questionnaire data through 23 March 2023 were included in the current analyses. Patients were 18 years or older, had a confirmed positive test for SARS-CoV-2 infection, were greater than 4 weeks post-acute infection, and were not fully recovered.

Recruitment for the EC began in October 2020. For this on-going randomized controlled clinical trial, patients were recruited from the greater Washington DC metropolitan area and attended study visits on-site to the NIH Clinical Center as outpatients. Patients had a confirmed SARS-CoV-2 infection that occurred at least 4 weeks prior to enrollment, and the presence of physical fatigue and/or physical limitations that stemmed from this infection. Persons with any medical or health ailments that increase the risk of exercise testing or training, affect the normal physiological response to exercise testing or training, or interfere with the ability to interpret the exercise testing data were excluded. Patients who underwent the CPET and completed VAS at the baseline visit through 31 December 2022 were included in the current analysis.

Recruitment for the ME/CFS and HV cohorts occurred between December 2016 and February 2020 (38). Of 484 ME/CFS inquiries, 217 individuals underwent detailed case reviews, with 27 ME/CFS and 25 HV performing research evaluations in-person. A further subgroup (9 ME/CFS and 9 HV) conducted the CPET and are presented in this study. Recruitment of HV was based on matching the demographics of ME/CFS patients, as much as possible. All ME/CFS patients met 2015 IOM ME/CFS criteria (4) and were confirmed by unanimous consensus by a panel of clinical experts. Since this cohort was recruited prior to the COVID pandemic, they are not confounded by SARS-CoV-2 vaccination or infection.

### 2.3 Measures and analyses

#### 2.3.1 Timing of data collection

For QC patients, Patient-Reported Outcomes Measurement Information System (PROMIS) questionnaires, 36-Item Short-Form Health Survey v2 (SF-36), Multidimensional Fatigue Inventory (MFI), and the DePaul Symptom Questionnaire (DSQ) subscale questions were collected within a month of enrollment. For EC patients, baseline measures including PROMIS, SF-36, and CPET were performed within a month of enrollment. CPETs were performed as outpatient visits for EC, and during inpatient stays of 10 days for ME/CFS and HV cohorts. EC, ME/CFS, and HV patients had visual analog scales for CFS symptoms (VAS) and qualitative interviews (QI) collected at several timepoints before and after CPET (prior to CPET, 15-min (EC only), 1-, 4-, 24-, 48-, and 72-h post-CPET). If patients were unable to respond at the exact time intervals, they were instructed to provide responses for how they felt at the respective timepoints. During the first 10 EC QI, patients reported little differences in physical, cognitive, and emotional symptoms across the timepoints. To reduce patient burden, the EC interview schedule was reduced to three timepoints: prior to CPET, 15-min post-CPET, and a single retrospective interview at the 72-h timepoint that reviewed each aforementioned timepoint.

#### 2.3.2 Questionnaires

##### 2.3.2.1 Self-assessment of functioning

###### 2.3.2.1.1 PROMIS

Patients in all cohorts completed PROMIS short forms for the domains of Fatigue, Pain Interference, Depression, Sleep Disturbance, and Anxiety. PROMIS is a validated and reliable system of measures, used to capture a wide range of patient-reported health status related to physical, mental, and social domains (39). These forms were identical across all cohorts with the exception of the Depression form with 1 of 8 items using slightly different wording for EC. PROMIS scores use T-score metrics (mean of 50 and a standard deviation of 10 based on the general population), and higher scores indicate worse severity of symptoms (40).

###### 2.3.2.1.2 SF-36

Patients from all cohorts completed the SF-36. The SF-36 is a validated questionnaire that reliably reflects health-related quality of life outcomes (41, 42). The Physical Component Summary (PCS) uses physical health components (e.g., Physical Functioning, Role-Physical, Bodily Pain, and General Health) to indicate physical limitations. The Mental Component Summary (MCS) uses various mental health components (e.g., Vitality, Social Functioning, Role-Emotional, and Mental Health) to indicate mental health status. Interpretation of component summary scores is norm-based, with a range spanning 25 to 60, where a composite score of 50 is equivalent to the mean of the general population, with a standard deviation equivalent to 10 (43).

###### 2.3.2.1.3 MFI

The ME/CFS, HV, and QC cohorts completed the MFI, a validated self-report instrument for assessing fatigue severity (44), including in the ME/CFS patient population (45). The 20 items are used to assess fatigue related to general, physical, emotional, and mental domains, with vigor providing an indication of the patient’s level of energy. Respondents used a scale (range of 1 to 5), and higher total scores are indicative of greater levels of fatigue.

##### 2.3.2.2 Self-assessment of PEM

###### 2.3.2.2.1 DSQ subscale

The QC and the ME/CFS cohorts filled in the DSQ (19). Five items from the DSQ are recommended for measuring PEM by the National Institutes of Health/Centers for Disease Control and Prevention Common Data Elements working group (19). Each question is scored for severity and frequency over 6 months. To have PEM, a person must have the symptom at least half the time and at least of moderate severity for any ONE symptom (16, 19). Patients in QC who indicated they were currently having fatigue or that fatigue was made worse by physical or mental activity (84% of QC patients) filled in the five DSQ items for current symptoms. As the QC cohort was designed to capture Long COVID patients early in their presentation, the 6-month frequency window of the DSQ could not be implemented. With this modification, any person reporting severe or worse on any of the five DSQ subscale items were classified as “Severe PEM,” those with at least one moderate symptom were classified as “Moderate PEM,” and anyone remaining (only mild or no symptoms on all questions, or who were not having fatigue and did not get asked the question) were classified as “No PEM.” Self-assessment of PEM in ME/CFS was based on the question “During the past month, how bad was your unusual fatigue after exertion?” from the CDC 2008 Symptom Inventory for CFS (CDC-SI) (46). ME/CFS patients also indicated either “yes or no” to the five items on the DSQ subscale.

###### 2.3.2.2.2 VAS

**Figure 2:**
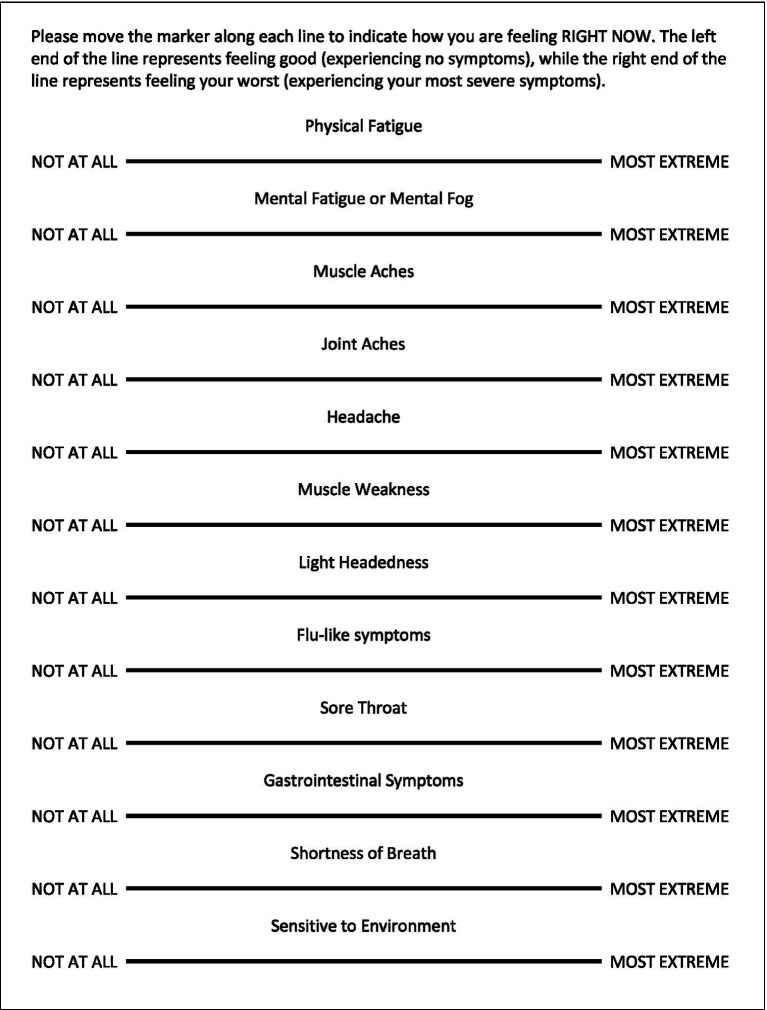
The VAS for CFS Symptom Questionnaire.

Patients in the EC, ME/CFS, and HV cohorts completed the VAS which included a total of 12 items for physical fatigue, mental fatigue/mental fog, muscle aches, joint aches, muscle weakness, lightheadedness, “flu-like” symptoms, headaches, sore throat, gastrointestinal discomfort, shortness of breath, and environmental sensitivity (Figure 2). Scales range from 0 to 100 with higher scores indicating increasing severity of the corresponding symptom. VAS data were analyzed for the four symptoms found most bothersome in our previous study (physical fatigue, mental fatigue, muscle ache, and headache) (37) as well as a composite VAS expressed as a sum of all 12 symptoms. Data from all three cohorts prior to CPET and 1-, 4-, 24-, 48-, and 72-h post-CPET were considered in the VAS analysis.

##### 2.3.2.3 Objective assessment of PEM following CPET

###### 2.3.2.3.1 QIs

QIs were found to effectively assess PEM in our previous study (37). A baseline level was established by conducting QI prior to performing the CPET. The QI included open-ended questions that invited patients to describe in their own words their current symptoms related to physical, cognitive, and emotional domains. These QIs were conducted by an experienced qualitative researcher, qualified healthcare practitioner, or clinical research member with experience in data collection and research measures. Importantly, all interviewers were trained to conduct the QI in a similar and consistent manner. In-person interviews were performed for all ME/CFS interviews, except for the 4-h post-CPET interview which was conducted via telephone due to patients being in a metabolic chamber. For EC patients, all interviews prior to and 15 min after the CPET were conducted in-person. Subsequent interviews were a mix of in-person or telephone depending on the patient’s location at the designated timepoints. Data from all three cohorts prior to CPET and 1-, 4-, 24-, 48-, and 72-h post-CPET were reported in the PEM QI analysis, with the additional 15 min after the CPET considered for the EC and the positive responses analysis.

**Figure 3:**
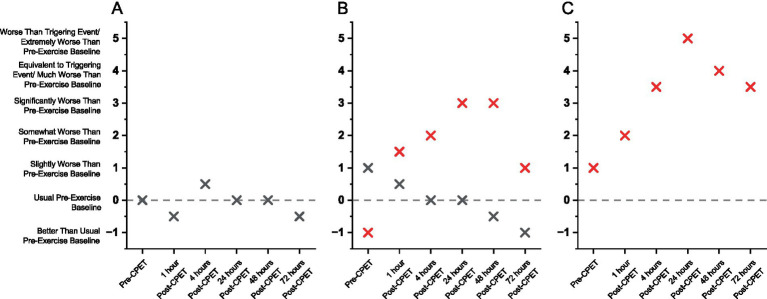
Graphical view of the QI among sample patients from **(A)** HV, **(B)** Long COVID EC, and **(C)** ME/CFS. Symbols denote severity of overall symptoms at specified timepoints in relation to the CPET. Red symbols denote patients meeting criteria for PEM. Dashed line indicates usual baseline levels. Two different patients are shown in (B) for EC.

A team of 4–6 researchers independently read and analyzed transcripts from EC, ME/CFS, and HV patients to assess PEM. Symptoms were independently plotted by each researcher for identifying the peak, trajectory, and most bothersome symptom for each patient. To achieve inter-rater reliability, transcripts were then reviewed together until consensus was reached. Disagreements were resolved by repeated in-depth discussions and line-by-line examination of transcripts. Once consensus was reached, the symptom severity at each timepoint was graphed, with the X-axis containing the varying time intervals and the Y-axis containing ticks describing the symptom severity. Completed qualitative data were then transformed into a quantitative format, where symptom severity was assigned a numerical value spanning −1.0 to 5.0, in 0.5 increments. Based on our validated methods (37), BOTH of the following criteria are necessary for a patient to have PEM: (1) reach at least *significantly worse* at peak (score of ≥3.0); and (2) must be above baseline at 24-, 48-, or 72-h post-CPET (i.e., cannot be considered fully recovered at 24 h). Figure 3 shows examples of trajectory after the CPET among the HV, EC, and ME/CFS cohorts. These quantitative data were then displayed in a heat map to visualize the differences in symptom severity between cohorts. Word clouds were created to display most bothersome symptoms across cohorts with font size proportional to frequency of the symptom having been identified as most bothersome by patients.

As many EC patients were observed to describe positive responses and endorsed “feeling better” following the CPET, a secondary review of transcripts was undertaken to capture the prevalence and scope of positive comments in the EC, ME/CFS, and HV cohorts. All transcripts for the follow-up timepoints after the CPET were systematically examined by four researchers independently for positive words or statements that could be directly attributed to the CPET. Neutral sounding statements and words (e.g., “I’m okay,” “feeling alright,” and “doing good”) were excluded, as were statements that could not be directly attributed to the CPET. Textual data were extracted and analyzed to identify emerging themes by four researchers using consensual qualitative research methods (47).

#### 2.3.3 Cardiopulmonary exercise test

A single CPET is effective for producing a robust PEM symptom flare in ME/CFS patients (12, 48, 49). The CPET was performed to volitional exhaustion on a treadmill in EC patients and a cycle ergometer for ME/CFS and HV. All ME/CFS patients were given a 15 W/min cycle ramp rate, while HV and EC patients had ramp rates chosen based on their estimated peak capacity for sex, age, and weight (50) and recent physical activity status. In all groups, gas exchange was measured breath-by-breath, with continuous monitoring of 12-lead ECG (CardiO2 Ultima; MedGraphics Corp, St. Paul, MN, USA) throughout the test. All calibrations were performed per manufacturer requirements prior to testing. Variables reported from the CPET include oxygen uptake (VO_2_), the ratio of expired carbon dioxide to oxygen consumed (VCO_2_/VO_2_) as the respiratory exchange ratio (RER), and heart rate (HR). Peak RER and VO_2_ were determined as an average of the last 20s achieved by the patient, prior to stopping due to volitional exhaustion. Peak VO_2_ was expressed as percent of predicted performance (50) and highest HR at peak as percent of predicted maximal HR based on age (i.e., 220-age).

### 2.4 Statistical analysis

**Figure 4:**
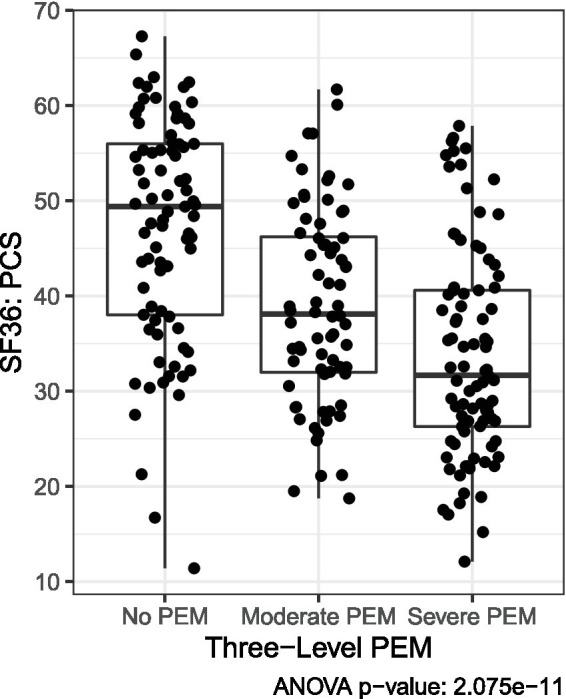
Three severity levels of PEM based on distribution of SF-36 PCS scores among Long COVID QC patients. As described in the methods, means were used to categorize Long COVID EC patients into PEM severity categories based on SF-36 PCS scores.

SF-36, PROMIS, and MFI scores were described across Long COVID and ME/CFS using mean and standard deviation and tested for group differences using analysis of variance (ANOVA). Pairwise Bonferroni-adjusted comparisons were conducted in significant ANOVAs. The characteristics of the HV group were also described separately. The EC protocol did not include the DSQ; therefore, the likelihood of PEM being reported among EC was estimated through a series of analyses investigating scales administered across both protocols. Using the three PEM severity groups detailed above (No PEM, Moderate PEM, and Severe PEM), the PEM group characteristics in the non-overlapping QC patients were described across PROMIS and SF-36 subscales to identify which scales had the most reliable correlation with PEM status based on ANOVA *p*-values. After identifying SF-36 PCS scale as having the best ability to differentiate between the three levels of PEM (Figure 4), EC individuals were classified into PEM groups by matching their SF-36 PCS score to the category where their score reduced the distance to the group mean.

Six patients were co-enrolled in QC and EC, presenting an opportunity to examine changes in responses over time and across studies. To further validate the estimation method above, we examined the within-subject correlations for PCS scores of co-enrollees in the QC and EC to examine whether the predicted EC PEM score correlated with the actual QC PEM score.

Heatmaps were constructed using OriginPro 2024 (OriginLab Corp, Northampton, MA) to visualize differences between individuals and cohorts across the specific timepoints of the CPET for the VAS and QI. Totals across the timepoints were used to arrange patients in ascending order within a cohort. The same minimal/maximal values, levels, and increments were used to display all cohorts.

## 3 Results

### 3.1 Patient demographics and functioning

Demographic characteristics for study patients are shown in Table 1. Like other studies on Long COVID and ME/CFS, patients were more commonly female (Female:Male; QC 3:1; EC 4:1). The QC had relatively equal representation across age groups, while 65% of the EC were 18–39 years of age. Both Long COVID cohorts were highly educated with >80% having a college or graduate degree. Two-thirds of the QC and 56% of the EC were > 7 months since symptom onset. No EC patient had disease severity greater than 4 on the World Health Organization COVID-19 ordinal clinical disease severity scale (51). Table 2 shows functioning levels across cohorts based on SF-36, PROMIS scales, and MFI. The ME/CFS cohort had significantly lower levels of physical functioning compared to either QC or EC, with QC also having significantly lower physical functioning compared to EC. The ME/CFS cohort self-reported the most pain interference (*M* = 62.6, SD = 9.1), followed by the QC ( = 55.2, SD = 10.5), and both had significantly more pain interference than the EC (*M* = 50.2, SD = 8.7). The QC reported significantly higher levels of depression and sleep disturbance compared to EC, with no difference in these domains between EC and ME/CFS. Fatigue was different across the cohorts; however, pairwise comparisons were not significant. Anxiety was the only symptom that did not differ between the cohorts. The HV cohort had better physical and mental functioning, as well as less symptom severity than expected for the general population. For MFI domains, HV had lower scores compared with ME/CFS and QC cohorts.

**Table 1:** Demographic characteristics across cohorts.

	QC	EC	ME/CFS	HV
N	244	34	9	9
Sex				
Female	184 (75.4)	27 (79.4)	5 (55.6)	6 (66.7)
Male	59 (24.2)	7 (20.6)	4 (44.4)	3 (33.3)
Not reported	1	–	–	–
Age				
18–29	27 (11.1)	10 (29.4)	3 (33.3)	2 (22.2)
30–39	69 (28.3)	12 (35.3)	1 (11.1)	2 (22.2)
40–49	53 (21.7)	4 (11.8)	3 (33.3)	2 (22.2)
50–59	54 (22.1)	5 (14.7)	1 (11.1)	3 (33.3)
60+	41 (16.8)	3 (8.8)	1 (11.1)	–
Race				
White	206 (84.4)	24 (70.6)	7 (77.8)	8 (88.9)
Other	38 (15.6)	10 (29.4)	2 (22.2)	1 (11.1)
Ethnicity				
Non-Hispanic	219 (89.9)	27 (79.4)	8 (88.9)	9 (100)
Hispanic	16 (6.6)	7 (20.6)	1 (11.1)	–
Unknown	9 (3.7)	–	–	–
Education				
Less than college degree	41 (16.8)	6 (17.6)	3 (33.3)	3 (33.3)
College degree	91 (37.3)	11 (32.4)	3 (33.3)	2 (22.2)
Graduate degree	111 (45.5)	17 (50.0)	3 (33.3)	4 (44.4)
Unknown	1	–	–	–
Marital status				
Married/living with partner	158 (64.8)	9 (26.5)	6 (66.7)	1 (11.1)
Never married	62 (25.4)	19 (55.9)	3 (33.3)	6 (66.7)
Divorced /Separated	21 (8.6)	5 (14.7)	–	2 (22.2)
Widowed	3 (1.2)	1 (2.9)	–	–
Time since symptom onset				
1–2 months	13 (5.3)	9 (26.5)	–	N/A
3–4 months	32 (13.1)	3 (8.8)	–	N/A
5–6 months	31 (12.7)	3 (8.8)	–	N/A
7–9 months	39 (16.0)	10 (29.4)	–	N/A
10–12 months	50 (20.5)	2 (5.9)	–	N/A
More than 12 months				
1–2 years	72 (29.5)	7 (20.6)	4 (44.4)	N/A
3–4 years	–	–	1 (11.1)	N/A
5–6 years	–	–	4 (44.4)	N/A
Unknown	7 (2.9%)	–	–	–

Data presented as n (%); – indicates none or data not collected.

**Table 2:** Self-reported physical and mental functioning across cohorts.

	ME/CFS (*n* = 9)	QC (*n* = 244)	EC (*n* = 34)	Between-group comparison F(df); *p*-value^a^	HV (*n* = 9)
SF-36					
Physical component score	21.27 (8.47)	39.8 (12.36)	46.01 (7.74)	15.8 (2, 284); *p* < 0.001 ME/CFS vs. QC p < 0.001 QC vs. EC *p* = 0.01 ME/CFS vs. EC *p* < 0.001	56.15 (3.18)
Mental component score	49.87 (7.06)	42.5 (11.17)	46.05 (9.50)	3.32 (2, 284); *p* = 0.04 No significant pairwise	56.70 (1.98)
PROMIS					
Fatigue	66.19 (4.62)	61.02 (10.72)	57.02 (8.62)	3.52 (2, 281); *p* = 0.03 No significant pairwise	36.76 (5.64)
Pain interference	62.60 (9.09)	55.19 (10.54)	50.23 (8.71)	6.08 (2, 281); *p* = 0.003 QC vs. EC *p* = 0.03 ME/CFS vs. EC *p* = 0.005	43.03 (4.63)
Depression^b^	48.18 (6.87)	54.19 (9.37)	48.44 (8.28)	7.27 (2, 279); *p* < 0.001 QC vs. EC *p* = 0.002	38.34 (5.61)
Sleep disturbance	53.11 (8.26)	54.98 (9.91)	50.17 (8.36)	3.74 (2, 281); *p* = 0.03 QC vs. EC *p* = 0.02	39.08 (5.20)
Anxiety	49.26 (4.39)	54.79 (9.46)	52.56 (8.13)	2.30 (2, 280); *p* = 0.10	39.66 (6.31)
MFI					
General fatigue	18.78 (1.62)	15.53 (4.10)	–		5.56 (1.33)
Physical fatigue	18.33 (1.49)	14.87 (4.75)	–		4.78 (0.63)
Reduced activity	17.33 (1.63)	13.86 (4.72)	–		6.0 (2.40)
Reduced motivation	10.56 (3.69)	11.73 (4.06)	–		4.67 (1.05)
Mental fatigue	14.67 (3.53)	13.29 (4.79)	–		6.0 (2.30)

Values shown as mean (± standard deviation), except for between-group comparison as noted; – indicates data not collected.

a *p*-value presented first overall, and those ANOVAs with significant global p-value were investigated for pairwise significance (Bonferroni adjusted).

b 1 of 8 questions had slight difference in wording for EC (Depression from PROMIS-57).

### 3.2 Prevalence of PEM based on self-assessments

#### 3.2.1 PEM questionnaire data

**Figure 5:**
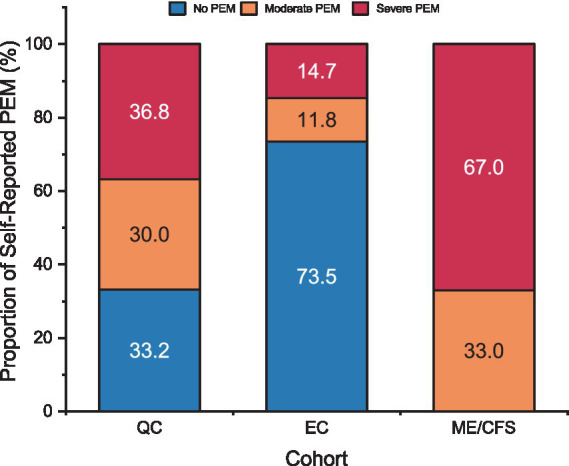
Proportion of patients by self-reported PEM severity for Long COVID QC, Long COVID EC, and ME/CFS. PEM categories represented for “No PEM” (blue), “Moderate PEM” (orange), and “Severe PEM” (red). Refer to methods for determination of PEM categories for each cohort.

Using the 3-level severity groupings of PEM described above, 66.8% of the QC self-reported as having PEM with 36.8% reporting severe PEM (Figure 5). Based on the estimation technique described above, a majority of the EC were expected to have No PEM (73.5%). Because our estimation method yielded lower prevalence of self-assessed PEM in Long COVID than seen in the literature (7, 20, 23), it is likely a conservative estimate. Among the remaining 26.5% of EC, 11.8% were expected to have Moderate PEM, and 14.7% to have Severe PEM. Two-thirds of ME/CFS patients reported severe fatigue after exertion with all having at least moderate fatigue. In addition, half of ME/CFS patients with severe fatigue after exertion endorsed all five DSQ subscale symptoms of PEM and 66% endorsed at least four of the symptoms.

#### 3.2.2 VAS

**Figure 6:**
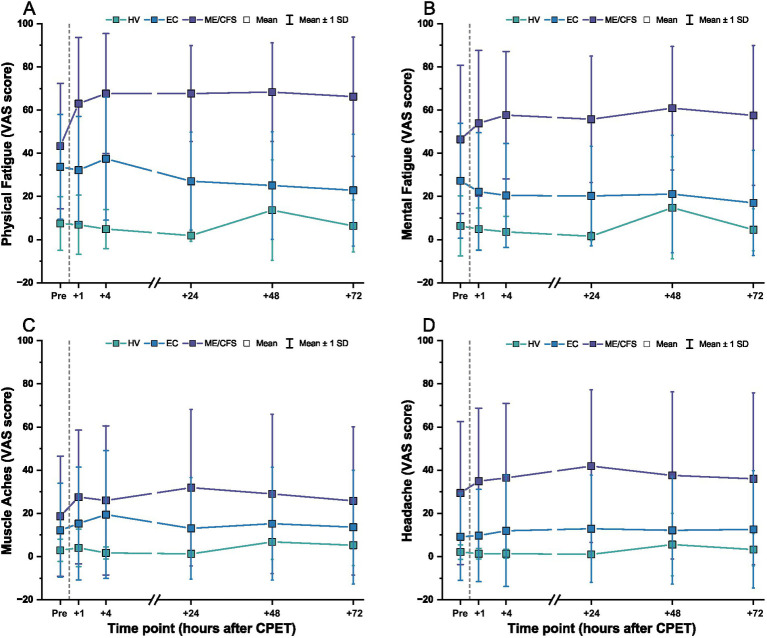
VAS scores before and after CPET for **(A)** physical fatigue, **(B)** mental fatigue, **(C)** muscle aches, and **(D)** headache for ME/CFS (purple), Long COVID EC (blue), and HV (green). Dashed line denotes when the CPET was performed. Symbols are mean ± one standard deviation.

Mean VAS scores for physical fatigue, mental fatigue, muscle aches, and headaches by cohort over time are displayed in Figure 6. As a group, higher symptoms were present in ME/CFS compared to EC or HV before the CPET and across all time points after CPET. Physical fatigue and muscle aches peaked at 4 h after the CPET in EC and declined over 72 h, while mental fatigue was lower after CPET across 72 h. Headaches in EC were generally stable throughout the timepoints. In contrast, HV had symptoms peak at 48 h after CPET, with generally minimal and stable symptoms reported after CPET. Heatmaps showing more granular data for individual patients are shown in Supplementary Figure S1.

**Figure 7:**
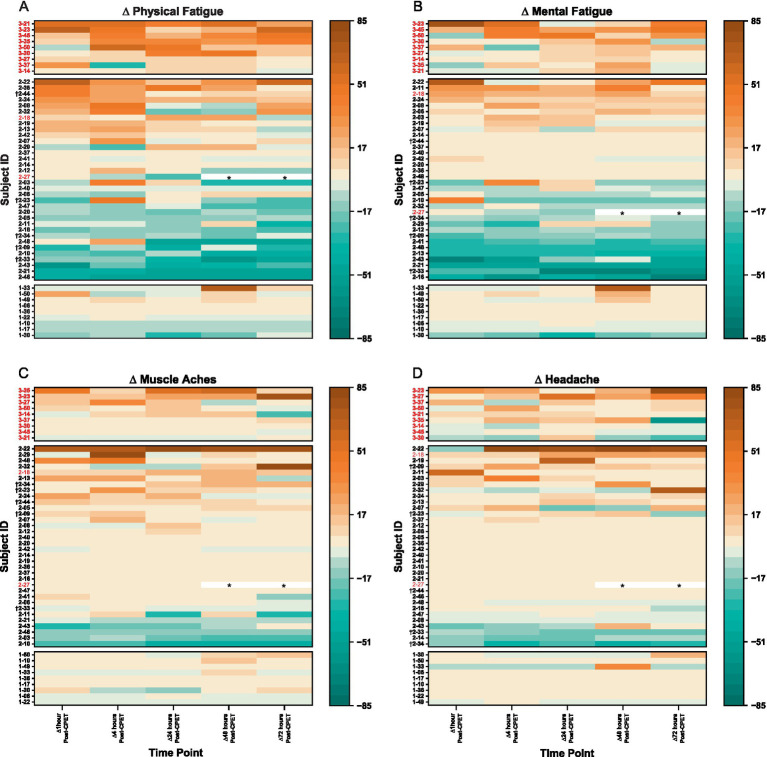
Heatmaps for change in VAS scores after CPET for **(A)** physical fatigue, **(B)** mental fatigue, **(C)** muscle aches, and **(D)** headache for ME/CFS (top section), Long COVID EC (middle section), and HV (bottom section). Time categories (x-axis) with individual patients (y-axis) are arranged in ascending order within respective group cohorts. Lighter pigments indicate minimal changes in VAS scores, with darker pigments depicting more (orange) or reduced (green) symptom severity from pre-CPET. Subject IDs in red met criteria for PEM by QI. Subject IDs denoted with (†) were in both EC and QC. Asterisks (*) denote data removed due to confounding variables.

When expressed as a change from pre-CPET baseline scores, VAS symptoms for ME/CFS became worse and were sustained over 72 h (Figure 7). This was especially observed for physical fatigue (range of change: *M* = +19.7, SD = 25.0 to *M* = +25.0, SD = 16.5) and mental fatigue (range of change: *M* = +10.6, SD = 26.7 to *M* = +17.6, SD = 9.8). In contrast, HV showed minimal change from pre-CPET baseline scores for these same domains over time [physical fatigue: *M* = −0.6, SD = 10.7 to *M* = +6.2, SD = 25.1; mental fatigue: *M* = −4.8, SD = 12.2 to *M* = +8.4, SD = 25.9]. For EC, lower mental fatigue was reported compared to pre-CPET scores across the 72 h (range of change: *M* = −10.2, SD = 23.3 to *M* = −5.0, SD = 26.3), with slight to minimal change observed for physical fatigue (range of change: *M* = −10.9, SD = 24.3 to *M* = +3.7, SD = 25.2). Change from pre-CPET baseline scores for composite VAS in EC was variable with some individuals reporting worsening of symptoms, minimal to no changes, or improvement of symptoms over time (range of change: *M* = −4.5, SD = 76.1 to *M* = +31.5, SD = 159.5) (Supplementary Figure S2). Interestingly, the EC patients identified as experiencing PEM were not consistently reporting severe symptoms. Notably, at the individual level, ~50% of EC patients improved across timepoints, particularly for physical and mental fatigue VAS, but improvement was only seen in ~33% of HV and not seen in ME/CFS.

#### 3.2.3 Objective assessment of PEM

##### 3.2.3.1 QI

**Figure 8:**
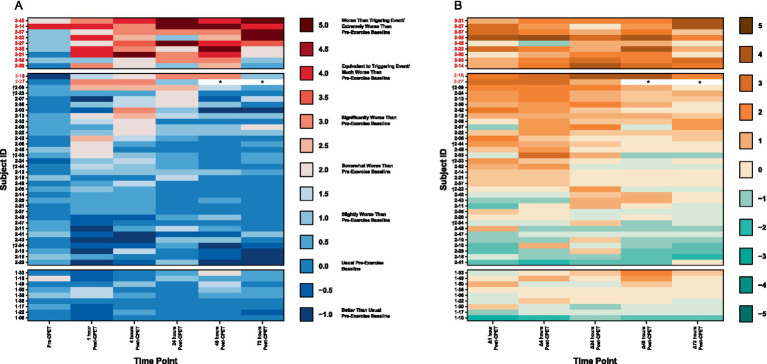
Heatmaps of **(A)** PEM based on QI and **(B)** change in symptom severity after CPET for ME/CFS (top section), Long COVID EC (middle section), and HV (bottom section). Time categories (x-axis) with individual patients (y-axis) are arranged in ascending order within respective group cohorts. Subject IDs in red met criteria for PEM by QI. Subject IDs denoted with (†) were in both EC and QC. Asterisks (*) denote data removed due to confounding variables.

All 9 ME/CFS patients, 2 of the 34 (5.9%) EC, and no HV met the threshold for PEM based on analysis of QI. The level of symptom severity reported in ME/CFS is more than seen in EC and HV (Figure 8). Nearly all EC patients report experiences similar to those of HV, with the exception that several EC patients report symptom improvements not noted among HV. A third of EC show symptom improvement that is not observed in ME/CFS. For EC patients with post-CPET symptoms, onset was generally immediate and peaking within 4 to 24 h, in contrast to symptom peaks seen at 48 to 72 h in ME/CFS.

##### 3.2.3.2 CPET outcomes

**Figure 9:**
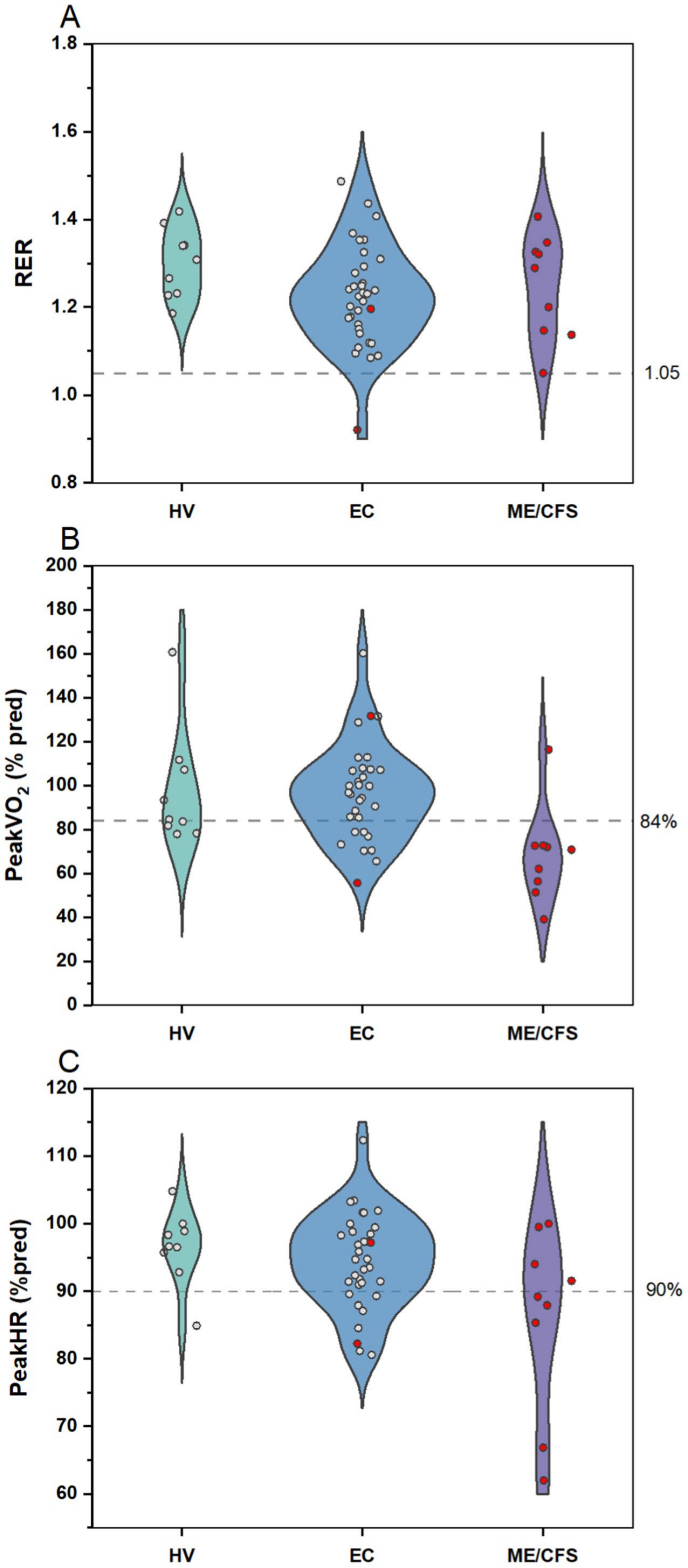
CPET outcomes for HV, Long COVID EC, and ME/CFS. Violin plots with distribution of individual data points for **(A)** RER, **(B)** peak VO_2_ as percent predicted, and **(C)** peak HR as percent of age-predicted maximal heart rate. Data points in red indicates patients meeting criteria for PEM by QI. Dashed line demarcates targets for **(A)** sufficient effort, **(B)** normal exercise capacity, and **(C)** expected peak HR on the CPET.

Patients in all cohorts gave sufficient effort on the CPET based on peak RER, with only one EC patient stopping early due to symptoms of dyspnea and one ME/CFS at 1.05 (Figure 9). However, only 11.1% of ME/CFS patients reached at least 84% of their predicted peak exercise capacity, suggesting lower than normal exercise tolerance for this group. In contrast, 55.6% of HV and 73.5% of EC patients reached their expected peak exercise capacity. A lower proportion of ME/CFS patients (44.5%) reached at least 90% of age expected maximal heart rate at peak exercise, compared to HV (88.9%) or EC (76.5%). Exercise capacity was relatively preserved among EC patients, with few meeting criteria for low exercise tolerance.

##### 3.2.3.3 Most bothersome symptoms across cohorts

**Figure 10:**
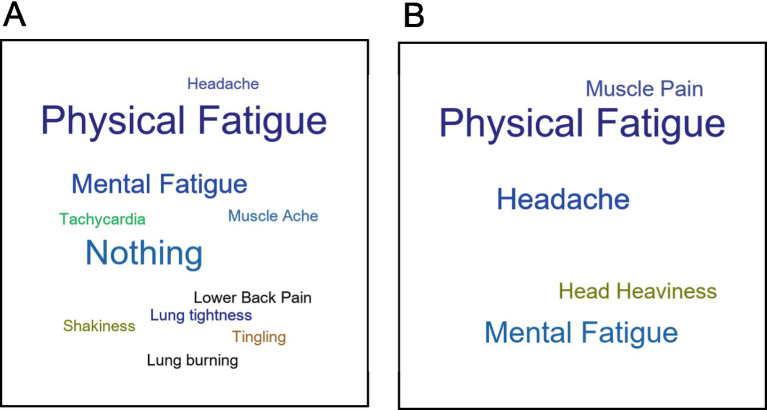
Word clouds of most bothersome symptoms from the QI for **(A)** Long COVID EC and **(B)** ME/CFS.

Word clouds displaying the most bothersome symptom after CPET for the EC and ME/CFS cohorts are shown in Figure 10 and reveal differences. While both cohorts had significant physical and mental fatigue, nine EC patients (26.5%) had no bothersome symptom indicated by “Nothing” in the figure. In addition, “lung tightness,” “lung burning,” “tachycardia,” “shakiness,” and “tingling” were unique to EC and were not among the most bothersome symptoms in ME/CFS.

#### 3.2.4 Co-enrolled patients

Six Long COVID patients were enrolled in both QC and EC, presenting an opportunity to examine changes in responses over time and across studies (Table 3). Patients ranged between 2 and 14 months from initial COVID infection, and majority performed QC prior to EC. Four patients self-assessed as having PEM based on the DSQ in the QC (1 moderate and 3 severe). However, for these patients, the QI indicated minimal or no symptoms both prior to CPET and by 24 h post-CPET (range: 0 to 2.5). For all four patients, symptoms either returned or improved compared to baseline by 72 h and none met the criteria for PEM by QI. Most of the patients reported moderate-to-severe physical dysfunction and fatigue on SF-36 and PROMIS-Fatigue, respectively, when measured in QC. Some interim improvement was noted when enrolled in EC, yet symptomatic impairment was still present. Five of the 6 patients performed the CPET in EC. All put forth a good effort (RER ≥ 1.05), and all but one reached >90% of age predicted maximal HR. Despite this, two patients demonstrated low exercise capacity with peak VO_2_ < 84% predicted. For these patients, low exercise tolerance was not related to fatigue severity or PEM. Within-subject correlations for SF-36 PCS scores of co-enrollees in the QC and EC reproduced well, within 5 to 10 points, except for one outlier. In summary, self-assessments for these patients did not align with objective assessments following the exercise stressor.

**Table 3:** Outcomes for 6 Long COVID patients enrolled in both studies (QC and EC).

Subject ID	COVID to QC (days)	QC to EC (days)	QC only	SF36 PCS			PROMIS Fatigue			CPET (EC only)			QI (EC Only)						
			PEM Severity	QC	EC	Diff	QC	EC	Diff	Peak RER	Peak VO_2_ (%pred)	Peak HR (%pred)	QI Pre-CPET	QI_1h Post-CPET	QI_4h Post-CPET	QI_24h Post-CPET	QI_48h Post-CPET	QI_72h Post-CPET	Meets PEM Criteria?
2–33	83	7	No PEM	53.2	45.5	-8	71.8	69.7	-2	1.2	99.9	89.6	0.5	2.0 **⇑**	1.0 **⇑**	0.5 **⇔**	0 **⇓**	0.5 **⇔**	No
2–44	184	41	No PEM	27.5	47.3	+20	53.3	41.0	−12	1.3	70.3	101.6	0	1.5 **⇑**	1.0 **⇑**	0.5 **⇑**	0 **⇔**	0 **⇔**	No
2–09	425	47	Mod PEM	44.5	47.7	+3	54.7	56.5	+2	1.3	78.9	93.2	0.5	2.5 **⇑**	2.5 **⇑**	2.5 **⇑**	1.5 **⇑**	0.5 **⇔**	No
2–34	74	52	Severe PEM	30.0	35.2	+5	74.4	65.7	−9	1.1	99.7	93.5	0	−0.5 **⇓**	−0.5 **⇓**	0.5 **⇑**	−1.0 **⇓**	−0.5 **⇓**	No
2–23	303	−43	Severe PEM	40.9	36.8	+4	77.7	66.5	+11	1.2	85.8	100.0	0.5	0.5 **⇔**	0.5 **⇔**	2.0 **⇑**	0 **⇓**	0 **⇓**	No
2–50	144	22	Severe PEM	32.6	26.9	−6	63.8	65.1	+1										

QC completed questionnaires only and EC completed questionnaires, CPET, and QI. Subject 2–50 did not participate in the CPET (screen fail). Subject 2–23 participated in EC prior to QC, therefore change in questionnaire scores reflects EC completed before QC. Refer to methods for determination of PEM severity for QC cohort. SF-36 scores for “Well below” or “Below” the general population is denoted in red and orange, respectively. PROMIS Fatigue scores for “Severe” or “Moderate” is denoted in red and orange, respectively. Difference in scores to indicate improvements or worsening of symptoms is denoted in green and red, respectively. Below expected normal values on the CPET are denoted in red. Arrows indicate direction of change from pre-CPET QI scores with increase symptoms (**⇑**), decrease symptoms (**⇓**), and no change in symptom (**⇔**).

#### 3.2.5 Positive responses following CPET

**Figure 11:**
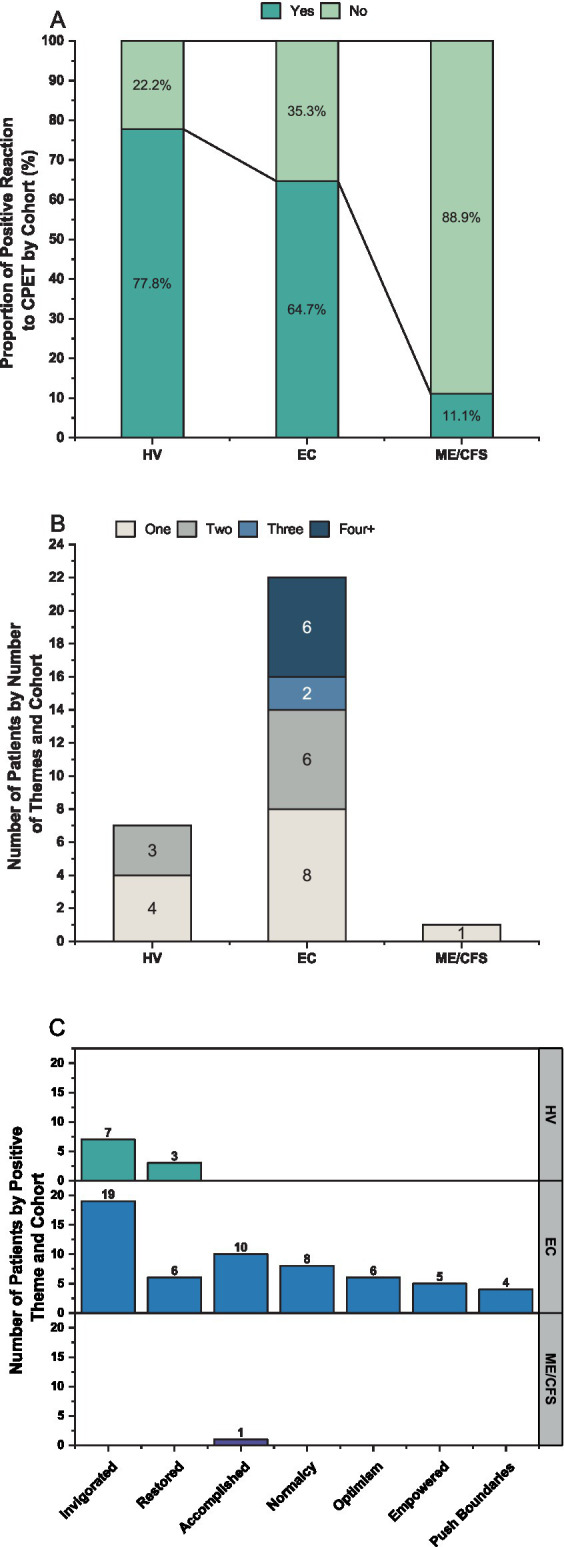
**(A)** Proportion of patients expressing a positive response after the CPET by cohort. **(B)** Number of patients by **(B)** number of positive themes and **(C)** theme category by cohort.

Analysis of positive responses to having performed the CPET revealed seven emergent themes (Figure 11). Most HV (77.8%) and EC patients (64.7%) expressed at least one theme compared to only one ME/CFS patient (11.1%). All themes were expressed by EC, while only two were seen by HV (Invigorated and Restored) and one in ME/CFS (Accomplished). Of the 22 EC patients who expressed a positive theme, 14 (63.6%) identified more than one. The most prevalent category for both the EC and HV was “Invigorated,” which includes feeling energized, alert, or supercharged following the CPET. “Restored” described a sense of renewal, feeling relaxed, or less stressed after the CPET. As one EC patient explained:

“I feel better. I was like very anxious and nervous before the test, but I feel a little more serene and calm now. I have never pushed myself since getting the infection at this level of exertion since recovering from the infection.”

“Accomplished” encompassed a sense of pride and achievement for completing the CPET and was described by one EC patient as

"In the last six months I haven’t felt so accomplished, so I’m really happy right now.”

Other patients used phrases indicating they felt back to their old self following the CPET, which were included in “Sense of Normalcy.” “Empowered” was used to incorporate overcoming fears, feeling motivated or confident from having performed the CPET. Statements that described feeling encouraged or hopeful for the future evoked the theme of “Optimism.” One EC patient described optimism as

“Well, just in general all symptoms included, I feel right now at this moment better and more encouraged than I have been since the beginning of this nightmare.”

Finally, comments describing testing their limits during the CPET were included under the theme “Pushing Boundaries.” Additional example quotes are shown in Table 4.

**Table 4:** Sample quotes for each of the seven positive themes.

Positive Theme	Sample quotes
Invigorated	“It was pretty good. I think that was like the peak maybe. I just felt pretty energized and like felt like I could get a lot of things done.”
Accomplished	“Oh, yeah. I’m proud that I accomplished the stress test without passing out. Yeah, I got through that. That was uncomfortable.”
Restored	“The power of exercise. I guess I got rid of some of whatever stress I was feeling during the walk.”
Sense of normalcy	“I actually feel great. The stress and I just feel more like my old self before COVID. I feel a lot like me, which is good. Haven’t felt this way in quite a long while.”
Empowered	“I’ve exerted myself moderately I think after Covid, because probably partly out of fear, because I wasn’t feeling well and from advice from my cardiologist and from doctors that did not want me to overexert too much when I was having more severe symptoms. But now that I do feel better, I feel good about it.”
Optimism	“I feel like there’s more hope for me; whereas, all these months since COVID I felt like in a hole that I just cannot get out and break on through to the other side, and I feel like that intense aerobic exercise, it put some light into everything for me mentally. Like I feel like there is hope.”
Pushing boundaries	“You know, I feel uplifted. Honestly, I just do, have done that just to have exercise, to have pushed myself and feel like I was able to do that, has been an emotionally uplifting experience. I feel very good.”

## 4 Discussion

### 4.1 Self- versus objective assessment of PEM in Long COVID

The current finding that 67% of the QC self-reported as having PEM with 37% reporting severe PEM is consistent with prior research finding high prevalence of self-reported PEM in Long COVID (7). Several recent studies from the Researching COVID to Enhance Recovery (RECOVER) initiative funded by the NIH, which includes thousands of participants throughout the U.S., have found high rates of self-reported PEM. For instance, Thaweethai et al. (52) found 87% of Long COVID patients self-reported PEM. Another recent study from RECOVER assessed ME/CFS symptoms in Long COVID patients, and PEM was the most frequently reported symptom, as measured by a single question, at 21.9% (53). A recent systematic review and meta-analysis evaluating the prevalence of PEM in Long COVID, which included 12 studies comprising 2,665 patients and seven self-report questionnaires, determined more than half reported PEM at 3 months post-infection (54). Objective QI assessment in the current small study found only 5.9% (2 out of 34) had PEM following CPET. These results suggest that specific exercise-related symptoms, when measured by self-report questionnaires, can be misclassified as PEM. None of the patients in EC, including those estimated to self-assess with PEM or who had PEM by QI, felt they could not continue with the study-related exercise training after completing the CPET, suggesting the test may have provided some reassurance.

The majority of EC patients were able to tolerate maximal exercise testing, with 26.5% demonstrating reduced exercise tolerance. Reductions in exercise capacity have been reported in those with Long COVID, compared to those with no lingering COVID symptoms (55), and previous studies have reported higher rates of exercise intolerance [between 32% (56) and 58.5% (57)] among those with Long COVID than observed in this study. Differences may be attributed to variations in study population (e.g., patients specifically referred for exercise testing, definitions of Long COVID) and resulting patient characteristics. For instance, patients in Norweg et al. (58) were mostly males in their 50s, with more than half being hospitalized during the acute illness, and 42% of the hospitalized patients required intensive care. In contrast, EC patients had mild COVID infections (less than 3% hospitalized) and the sample consisted of primarily younger (mean 39 years) and female (79%) compared to other studies. Appelman et al. (6) observed substantially lower maximal oxygen uptake among Long COVID patients meeting DSQ criteria for PEM compared to controls that recovered from COVID with no residual symptoms; however, it was not noted what percent of predicted normal peak values were observed for individual subjects. Previous studies reporting on exercise intolerance in Long COVID (56–60) did not report or assess symptoms following the CPET. While exercise intolerance has been associated with PEM in Long COVID, the association between lower exercise capacity and PEM has not been well studied.

### 4.2 PEM in Long COVID versus ME/CFS

In the current study, PEM in ME/CFS was more prevalent and severe than in Long COVID. While all nine ME/CFS patients were found to have PEM following CPET, only 5.9% of the 34 Long COVID patients and no HV developed PEM. Furthermore, the ME/CFS cohort had reduced exercise tolerance compared to the EC and HV. While physical and mental fatigue were seen as most bothersome symptoms in both ME/CFS and Long COVID, all ME/CFS patients had reported a most bothersome symptom while more than a quarter of EC patients had none.

While some prior research has found similarities between PEM in Long COVID and ME/CFS, others have also found notable differences. A study comparing self-reported PEM in Long COVID patients and ME/CFS found the two groups presented similarly (7). That study compared PEM questionnaire data from patients seeking care for Long COVID at a single care center to patients diagnosed with ME/CFS who were already enrolled in a separate study. They found that 79 of 80 Long COVID patients had PEM and that onset and recovery time was similar between the two groups. They also saw significantly more sleepiness, respiratory issues, depression and anxiety, irregular body temperature, and excessive thirst in Long COVID than ME/CFS, which they hypothesized could be due to ME/CFS patients having had longer to learn and manage triggers of PEM. Another study surveyed Long COVID and ME/CFS patients on an extensive list of symptoms and found ME/CFS patients were more symptomatic than Long COVID in all domains except orthostatic (61). That same study team later found that PEM improved significantly in Long COVID patients over the course of 1 year, but no improvement was seen for ME/CFS patients (34). Current findings suggest that objective assessment of PEM might uncover nuances between patient populations not readily apparent with self-assessment.

### 4.3 Positive responses to CPET in Long COVID

A main finding in the current study was that the majority of Long COVID patients had positive responses following the CPET, which is consistent with the observation by Laguarta-Val (28) of positive reactions accompanying exercise among Long COVID patients. Importantly, while several EC patients reported improvement of symptoms after performing the CPET, this was rarely seen among ME/CFS. Furthermore, both EC and HV endorsed the category of Invigorated most frequently, suggesting that exercise responses for many EC patients are more representative of HV than ME/CFS. Two other impactful positive themes include the categories of Empowered and Normalcy which were mentioned by 17.6 and 23.5% of EC patients, respectively. Patients who felt “empowered” mentioned overcoming “fear of overexertion,” “pushing myself for the first time,” “I know now that I can do it,” and “realizing that if I did that stress test…I can go on longer walks and maybe…hike again.” Normalcy encompassed “the best that I’ve felt since having the COVID,” “I just feel more like my old self before COVID,” and “it totally feels 100 percent like a pre-COVID day. I feel completely back.” These were also reflected in the physical and mental fatigue VAS, where EC patients reported symptom improvements after the CPET.

### 4.4 Implications

As presented in this small study, the presence of PEM may be over-reported in Long COVID patients, especially if relying on self-report questionnaires alone. Thereby, performance of a single CPET, coupled with monitoring in the days after for potential symptom development/worsening and formal assessment of PEM by QI, may have value for improved discrimination of those with Long COVID that may benefit from targeted exercise training therapies. This could mitigate the risks of applying an overly cautious approach to those that may not have PEM and could benefit from more traditional exercise training recommendations. Further studies are clearly needed to understand the value of a single CPET in guiding exercise interventions for optimizing outcomes among Long COVID.

### 4.5 Limitations

The current study had several limitations. Due to small sample sizes in the ME/CFS and HV cohorts, only a narrow range of symptoms were identified as most bothersome, which may not be representative of the ME/CFS population as a whole. Patients recruited for the QC and EC were convenience samples and could have had more or less severe Long COVID presentations than the general population. Patients with medical conditions that increase the risk of exercise testing were excluded from the EC yielding a homogenous study population. It is also possible that selection biases impacted recruitment such that individuals with Long COVID who self-selected to participate in an exercise-focused clinical trial may also be younger and less avoidant to exercise than the general population of Long COVID patients. Seven EC (21%) and 13 QC (5.3%) patients were < 3 months from their acute infection and outside the window of some definitions of Long COVID; it is unknown whether symptoms would have spontaneously improved over time. Patients in the ME/CFS study stayed in the NIH Clinical Center for 4 to 5 days following CPET, while EC patients performed CPET as outpatients. Although ME/CFS patients were away from any stressors in their daily lives, the procedures they underwent during the 3 days following CPET may have impacted PEM symptoms. Similarly, activities EC patients undertook following CPET in their daily lives may have exacerbated symptoms. Finally, the HVs in the current study were recruited to match the ME/CFS, not Long COVID patients with respect to demographics. Despite these limitations, the rigorous and consistent methodology used for assessment of PEM across cohorts provides assurance in the findings.

## 5 Conclusion

Long COVID patients in the current small study self-assess as having PEM at high rates. Yet in a subset that performed a standardized exercise stressor, few were found to have PEM through objective assessment, and many associated the exercise stressor with positive responses. When comparing PEM in ME/CFS vs. Long COVID in the current study, PEM in ME/CFS is more severe and without corresponding positive responses. The use of a single provocative exercise test with tracking of potential symptom development and objective assessment of PEM may provide useful information for guiding exercise recommendations in Long COVID patients.

## Data Availability

The original contributions for ME/CFS data presented in the study are publicly available. This data can be found here https://mecfs.rti.org/research/. The Long COVID datasets presented in this article are not readily available because they are part of on-going clinical studies. Requests to access the datasets should be directed to brian.walitt@nih.gov.

## Footnotes

^1^ www.clinicaltrials.gov

## Supplementary material

SUPPLEMENTARY FIGURE S1 Heatmaps for VAS scores before and after CPET for **(A)** physical fatigue, **(B)** mental fatigue, **(C)** muscle aches, and **(D)** headache for ME/CFS (top section), Long COVID EC (middle section) and HV (bottom section). Time categories (x-axis) with individual patients (y-axis) are arranged in ascending order within respective group cohorts. Brighter pigments indicate higher VAS scores and endorsement of more severe symptoms. Subject IDs in red met criteria for PEM by QI. Subject IDs denoted with (†) were in both EC and QC. Asterisks (*) denote data removed due to confounding variables.

SUPPLEMENTARY FIGURE S2 **(A)** Composite VAS scores before and at multiple time points after CPET for ME/CFS (purple), Long COVID EC (blue) and HV (green). Dashed line denote when the CPET was performed. Symbols are mean ± one standard deviation. **(B)** Heatmap for composite VAS scores before and after CPET for ME/CFS (top section), Long COVID EC (middle section) and HV (bottom section). Time categories (x-axis) with individual patients (y-axis) are arranged in ascending order within respective group cohorts. Brighter pigments indicate higher VAS scores and endorsement of more severe symptoms. Subject IDs in red met criteria for PEM by QI. Subject IDs denoted with (†) were in both EC and QC. Asterisks (*) denote data removed due to confounding variables. **(C)** Heatmap for change in composite VAS scores after CPET for ME/CFS (top section), Long COVID EC (middle section) and HV (bottom section). Time categories (x-axis) with individual patients (y-axis) are arranged in ascending order within respective group cohorts. Lighter pigments indicate minimal changes in VAS scores, with darker pigments depicting more (orange) or reduced (green) symptom severity from pre-CPET. Subject IDs in red met criteria for PEM by QI. Subject IDs denoted with (†) were in both EC and QC. Asterisks (*) denote data removed due to confounding variables.
