# Supplementary figures and images for: Post-exertional malaise in Long COVID: subjective reporting versus objective assessment
Source: Front Neurol. 2025 Apr 23;16:1534352. doi: 10.3389/fneur.2025.1534352 (PMC12055772; doi:10.3389/fneur.2025.1534352)

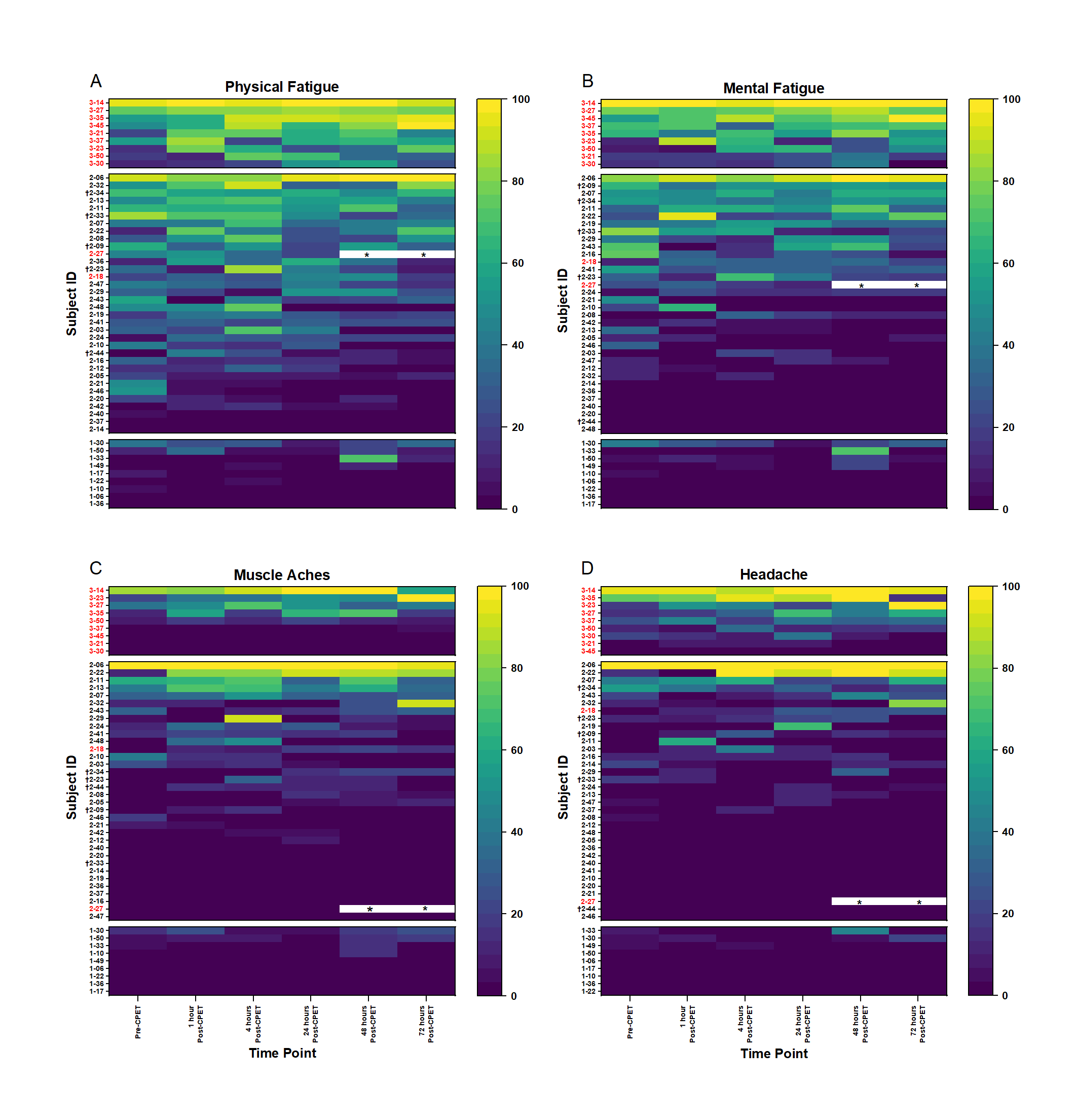

Supplement: SUPPLEMENTARY FIGURE S1 — Heatmaps for VAS scores before and after CPET for (A) physical fatigue, (B) mental fatigue, (C) muscle aches, and (D) headache for ME/CFS (top section), Long COVID EC (middle section) and HV (bottom section). Time categories (x-axis) with individual patients (y-axis) are arranged in ascending order within respective group cohorts. Brighter pigments indicate higher VAS scores and endorsement of more severe symptoms. Subject IDs in red met criteria for PEM by QI. Subject IDs denoted with (†) were in both EC and QC. Asterisks (*) denote data removed due to confounding variables. [file Image_1.tif]

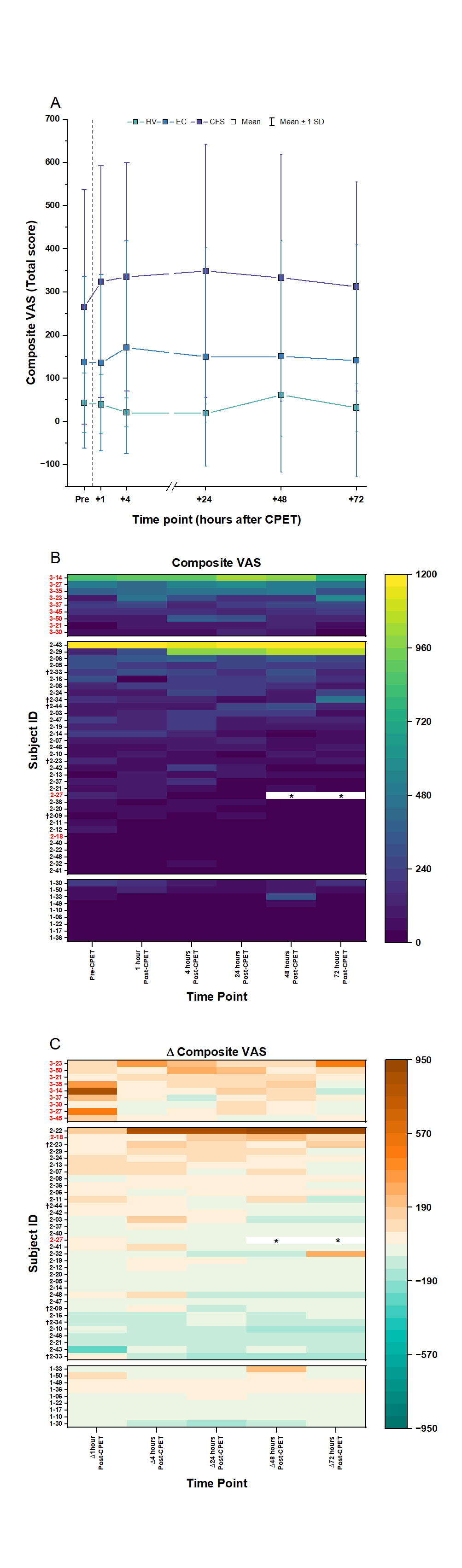

Supplement: SUPPLEMENTARY FIGURE S2 — (A) Composite VAS scores before and at multiple time points after CPET for ME/CFS (purple), Long COVID EC (blue) and HV (green). Dashed line denote when the CPET was performed. Symbols are mean ± one standard deviation. (B) Heatmap for composite VAS scores before and after CPET for ME/CFS (top section), Long COVID EC (middle section) and HV (bottom section). Time categories (x-axis) with individual patients (y-axis) are arranged in ascending order within respective group cohorts. Brighter pigments indicate higher VAS scores and endorsement of more severe symptoms. Subject IDs in red met criteria for PEM by QI. Subject IDs denoted with (†) were in both EC and QC. Asterisks (*) denote data removed due to confounding variables. (C) Heatmap for change in composite VAS scores after CPET for ME/CFS (top section), Long COVID EC (middle section) and HV (bottom section). Time categories (x-axis) with individual patients (y-axis) are arranged in ascending order within respective group cohorts. Lighter pigments indicate minimal changes in VAS scores, with darker pigments depicting more (orange) or reduced (green) symptom severity from pre-CPET. Subject IDs in red met criteria for PEM by QI. Subject IDs denoted with (†) were in both EC and QC. Asterisks (*) denote data removed due to confounding variables. [file Image_2.tif]
